# Statistical Scene-Based Non-Uniformity Correction Method with Interframe Registration

**DOI:** 10.3390/s19245395

**Published:** 2019-12-06

**Authors:** Baolin Lv, Shoufeng Tong, Qiaoyuan Liu, Haijiang Sun

**Affiliations:** 1 School of Opto-Electronic Engineering, Changchun University of Science and Technology, Changchun 130033, China; tsf1998@sina.com; 2 Changchun Institute of Optics, Fine Mechanics and Physics, Chinese Academy of Sciences, Changchun 130033, China; liuqy@ciomp.ac.cn (Q.L.); sunhaijiang@126.com (H.S.)

**Keywords:** statistical scene, non-uniformity correction, fixed-pattern noise, adaptive, registration method

## Abstract

The non-uniform response in infrared focal plane array (IRFPA) detectors inevitably produces corrupted images with a fixed-pattern noise. In this paper, we present a novel and adaptive scene-based non-uniformity correction (NUC) method called Correction method with Statistical scene-based and Interframe Registration (CSIR), which realizes low delay calculation of correction coefficient for infrared image. This method combines the statistical method and registration method to achieve a better NUC performance. Specifically, CSIR estimates the gain coefficient with statistical method to give registration method an appropriate initial value. This combination method not only reduces the need of interactive pictures, which means lower time delay, but also achieves better performance compared to the statistical method and other single registration methods. To verify this, real non-uniformity infrared image sequences collected by ourselves were used, and the advantage of CSIR was compared thoroughly on frame number (corresponding to delay time) and accuracy. The results show that the proposed method could achieve a significantly fast and reliable fixed-pattern noise reduction with the effective gain and offset.

## 1. Introduction

Infrared focal plane array (IRFPA) sensors are commonly used in military and civilian applications such as disaster assessment, security monitoring [1], medical treatment, forest fire prevention, national defense [2], etc. Influenced by factors such as sensor fabrication process, IRFPA often receives serious non-uniform noise, which affects the imaging quality of the system [3]. In addition, this bottleneck problem restricts the infrared detection system [4]. The non-uniformity of an IRFPA is also called fixed pattern noise (FPN). To solve this problem, various NUC techniques [5,6,7,8] have been proposed to improve the quality of images produced.

NUC methods are mainly divided into two categories: calibration-based correction methods (CBNUC) [9] and scene-based correction methods (SBNUC) [10]. Calibration-based methods mainly include single-point correction [11] and two-point correction [12]. Both methods are based on a linear model and require less computation. The most commonly used two-point method in engineering only needs one multiplication and one addition to realize real-time non-uniformity correction. This method calculates the gain and offset coefficients by using the blackbody responses at two different temperatures. However, when the calibrated temperature is far from the reference temperature point, the correction error increases gradually. In addition, CBNUC cannot solve the problem of radiation response drift; thus, it has to be repeated by inserting a uniform radiation source into the view, which affects the quality of images produced.

To address the above disadvantage, many SBNUC methods were proposed [13,14]. The SBNUC methods can adaptively alleviate FPN fluctuation by using scenario information without a uniform radiation source. Therefore, the scene-based method can correct the optical system, and update the gain and offset coefficient in real-time so as to solve the problem of radiation drift. Current SBNUC methods can be divided into three main approaches: the statistical methods [15], the registration methods [16], and the neural network methods [17].

Statistical methods can make good use of the spatial information from the focal plane of each detector, and then fine tune the gain and offset to achieve NUC. The LMS (Least mean square) method [18] proposed by Hayat is based on the linear model; the gain and offset are estimated by an infinite impulse response (FIR) filter, and are then adaptively updated via a Wiener filter. However, it is easy to generate ghosts when the scene changes slightly. Geng proposed a correction method for interpolation statistics of adjacent pixels [19]. In this method, the non-uniformity of focal plane is modeled as horizontal and vertical non-uniformity, the correction coefficients are estimated separately, and then the final correction coefficients are synthesized. The disadvantage of this method is that pre-calibration based on blackbody method is needed, and a slight ghost residual remains. These methods can correct the images in relatively quickly and have small storage demands.

Compared with statistical methods, registration-based correction method is more reasonable and converges more quickly [20]. This method assumes that the response of each pixel in the same scene is relatively consistent within several adjacent frame intervals. Zuo proposed an interframe least mean square (IRLMS) method [21] and utilized a phase-correlation method for motion estimation. The images produced have almost no ghosting artifacts. However, the registration-based methods are not practical enough due to their high computational complexity and large storage demands, because they need many pictures to achieve good performance.

In addition, the neural network-based SBNUC methods receive extensive attention because they not require high linearity and stability of detector coefficients. Hardie [22] proposed a deghosting method based on a temporal gate in the neural network; by gating the update coefficients, the accumulation of incident radiation estimation errors during long motion paused is avoided. This method has been widely studied due to its good performance. However, this kind of methods require a large amount of research work and computation in application, which means it is hardly used in high real time situation.

Based on the analysis of the advantages and disadvantages of existing methods, this paper proposes a hybrid correction method based on both statistics and registration. We use the statistical method to get gain coefficients and then use these gain coefficients as the initial values for registration methods to obtain offset coefficients more rapidly and accurately. In this way, both the spatial information between pixels and temporal motion estimation are considered in the process of non-uniform correction. Specifically, a block-based correction method based on statistical consistency of adjacent pixels is used to obtain gain coefficients and a registration method utilizing the phase-correlation algorithm that minimizes the mean square error between two properly aligned images is used to obtain the offset coefficient.

This paper is organized as follows. The related work is introduced in Section 2. Section 3 presents the detailed analysis of the proposed correction algorithm. Experimental results and comparative study of related methods are presented in Section 4. It is worth mentioning that different methods were tested on the real infrared sequences collected by ourselves. The experimental results show the superiority of our proposed method. Finally, the conclusion is presented in Section 5.

## 2. Non-Uniformity Correction

### 2.1. Non-Uniformity Observation Model

Suppose I is an image with non-uniformity noise, the output after NUC correction is J. For the pixels (i, j) in the image before and after correction, we would always consider them in a linear model [23] as follows:

(1) $$J_{i,j}\left(f\right){=k}_{i,j}\left(f\right)\cdot I_{i,j}\left(f\right){+o}_{i,j}\left(f\right)$$

where $J_{i,j}\left(f\right)$ and $I_{i,j}\left(f\right)$ are the gray values of pixel (i, j) before and after correction, respectively. $k_{i,j}\left(f\right)$ and $b_{i,j}\left(f\right)$ are the real gain and offset correction coefficients, respectively. We assume that the gains and offsets would drift vary slowly and slightly in time when compared with other inevitable errors in infrared detectors [18,19]. When detectors work in high frame rate, high speed scanning mode, the typical scanning rate is 10°/s, and the frame rate is 100–400 Hz. In this situation, with in 1–10 s time, $k_{i,j}\left(f\right)$ and $b_{i,j}\left(f\right)\;$ could be regarded as the same, thus they could share the same subscript n . Therefore, the equation above can be re-written as follows:

(2) $$J_{n}\left(f\right){=k}_{n}\left(f\right)\cdot I_{n}\left(f\right){+o}_{n}\left(f\right)$$

The main purpose of the NUC algorithm is to estimate the value of $I_{n}\left(f\right)$ accurately. Using Equation (2), the value of $I_{n}\left(f\right)$ can be obtained by the following equation:

(3) $$I_{n}\left(f\right){=w}_{n}\left(f\right)\cdot J_{n}\left(f\right){+b}_{n}\left(f\right)$$

where the new parameters $w_{n}\left(f\right)$ and $b_{n}\left(f\right)$ are, respectively, the gain and offset of the linear correction model. Their relation with the real gain and offset can be represented as follows:

(4) $$w_{n}\left(f\right)=\frac{1}{k_{n}\left(f\right)}$$

(5) $$b_{n}\left(f\right)=\frac{o_{n}\left(f\right)}{k_{n}\left(f\right)}$$

### 2.2. Non-Uniformity Correction Method Based on Inter-Frame Registration

In the correction method based on inter-frame registration [21], there is firstly an error function defined as follows:

(6) $${\;e}_{n}\left(i,j\right){=I}_{n-1}\left({i-d}_{i}{,j-d}_{i}\right){-I}_{n}\left(i,j\right)$$

which is defined as the corresponding difference between the two adjacent corrected frames. Moreover, $d_{i}{,d}_{j}$ is the translation between two adjacent frames. Substituting Equation (3) into Equation (6), we have

(7) $$e_{n}\left(i,j\right)=\left(w_{n}\left({i-d}_{i}{,j-d}_{i}\right)\cdot J_{n-1}\left({i-d}_{i}{,j-d}_{i}\right){+b}_{n}\left({i-d}_{i}{,j-d}_{i}\right)\right){-(w}_{n}\left(i,j\right)\cdot J_{n}\left(i,j\right){+b}_{n}\left(i,j\right))$$

The purpose of inter-frame registration is to minimize the error $e_{n}\left(i,j\right)$ in the mean square error sense, thus the objective function E should be

(8) $$E\left(i,j\right)=\underset{n}{\sum }e_{n}{\left(i,j\right)}^{2}=\underset{n}{\sum }{(\left(w_{n}\left({i-d}_{i}{,j-d}_{i}\right)\cdot J_{n-1}\left({i-d}_{i}{,j-d}_{i}\right){+b}_{n}\left({i-d}_{i}{,j-d}_{i}\right)\right){-(w}_{n}\left(i,j\right)\cdot J_{n}\left(i,j\right){+b}_{n}\left(i,j\right)))}^{2}$$

At this time, w and b need to be updated recursively to minimize the objective function, to realize NUC. Generally, the gradient-decent strategy is employed

(9) $$w_{n+1}\left(i,j\right)=\left\{\begin{array}{c} w_{n}\left(i,j\right)+\rho \cdot e_{n}\left(i,j\right)\cdot J_{n}\left(i,j\right),\;\mathrm{overlapped}\;\mathrm{region}\\ w_{n}\left(i,j\right),\;\mathrm{otherwise} \end{array}\right.$$

(10) $$b_{n+1}\left(i,j\right)=\left\{\begin{array}{c} b_{n}\left(i,j\right)+\rho \cdot e_{n}\left(i,j\right),\;\mathrm{overlapped}\;\mathrm{region}\\ b_{n}\left(i,j\right),\;\mathrm{otherwise} \end{array}\right.$$

where ρ is the learning rate. Moreover, the overlap region is the overlapped area between adjacent frames. It can be seen from the above formula that we could have k results (w and b) when using k pictures. In practice, we could use these k results to correct the corresponding image, use the average value of these k results (w and b) calculated by previous images to correct the latest pictures in real time, and use the latest pictures to updated the result constantly. In this way, we could correct the infrared pictures in real time and the number of pictures used in registration method decides the delay time of correction coefficient calculation. The lower is the number of pictures needed, the lower is the time delay.

As with other optimization algorithms, using fewer pictures means lower convergence rate. Many methods can achieve lower convergence rate and the easiest way is to use appropriate initial values of w and n. Generally, consistent w (all-one) and b (all-zero), or w and b corresponding to two points correction results are used as initial values for the registration method. However, these initial values need many frames to achieve relatively low NUC error because the correction results might not be appropriate for the current scenario [15], which was also proved in our experiments. To achieve lower frame number and higher NUC correction accuracy, we propose a statistical method that is more appropriate for the current scenario to calculate the initial values.

### 2.3. Non-Uniformity Correction Method Based on Consistency of Adjacent Pixels

The statistical methods usually achieve NUC by only correcting the gain coefficient. In the NUC method based on consistency of adjacent pixels [19], it is considered that each pixel is closest to its nearest four pixels, thus the image is statistically consistent with similar scenes, which could be described as follows:

(11) $${\mathrm{mid}}_{F}\left[\frac{J_{i,j}\left(f\right)}{\sqrt[{4}]{J_{i-1,j}\left(f\right)J_{i+1,j}\left(f\right)J_{i,j-1}\left(f\right)J_{i,j+1}\left(f\right)}}\right]=1$$

where ${\mathrm{mid}}_{F}[.]$ is used to calculate the median of F values. In this case, the adjacent four pixels can be reduced to two, because the non-uniformity exists in the horizontal and vertical directions of the array detector. Thus, considering Equation (2), the above formula can be simplified as follows:

(12) $$\frac{k_{i,j}}{\sqrt{k_{i-1,j}k_{i,j-1}}}{\mathrm{mid}}_{F}\left[\frac{I_{i,j}\left(f\right)}{\sqrt{I_{i-1,j}\left(f\right)I_{i,j-1}\left(f\right)}}\right]=1$$

For the convenience of formula deduction, the ratio between a pixel and its adjacent pixels is defined as

(13) $$r_{i,j}\left(f\right)=\frac{I_{i,j}\left(f\right)}{\sqrt{I_{i-1,j}\left(f\right)I_{i,j-1}\left(f\right)}}$$

Setting the median of $r_{i,j}$ in F frames as ${\;r\;˜}_{i,j}{=\mathrm{mid}}_{F}{[r}_{i,j}\left(f\right)]$, the gain coefficients can be obtained using Equations (11) and (12):

(14) $$k_{i,j}=\frac{\sqrt{k_{i-1,j}k_{i,j-1}}}{{\;r\;˜}_{i,j}}$$

Obviously, this method uses iteration to calculate the gain coefficient of any big error in the picture sequence, which is caused by uncorrected flash pixel or occasional large shot noise that can lead to error transfer. As a result, the proposed statically method itself is not good enough. However, when combined with the registration method, the accuracy can be improved, which was proved in the experiments. Overall, the gain coefficient is obtained by recursion as follows.

## 3. Proposed Method

**Figure 1:**
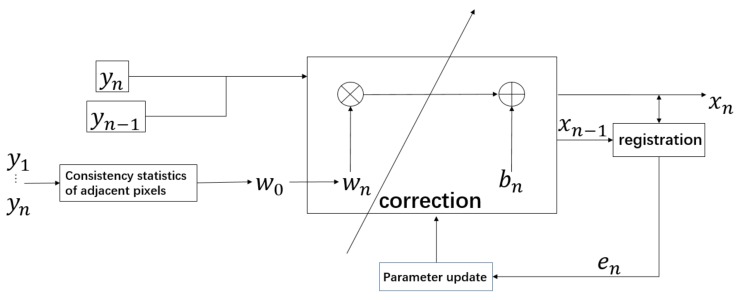
Scheme of the proposed statistical scene-based non-uniformity correction method with interframe registration (SIR).

In the registration-based method, there is usually no research on the initial value of parameters, and the results of NUC mainly depend on the effectiveness of the registration. The statistical method’s purpose is to obtain the gain coefficient; it only processes the spatial information of single image, ignoring the sequential information between frames. Therefore, our paper innovatively combines registration-based and statistical correction-based methods together. The overview of our algorithm is presented in Figure 1.

In the registration-based correction methods, the initial values of gain and offset coefficients are important for iterative convergence. Therefore, setting an optimal initial correction coefficient is the key factor for obtaining the result. If the coefficients can be set as appropriate values before iteration, the time for iteration would be shortened and the convergence value would be more accurate. In the method of IRLMS, the initial gain is set as an all-one matrix and the initial offset is set as an all-zero matrix. Different from this, our approach generates the initial gain coefficient by a modular pixel consistency method for the registration-based NUC method.

### 3.1. Modular Pixel Consistency Correction Method

The modular-based pixel consistency NUC method is mainly used to obtain the initial gain coefficient by analyzing the spatial relationship between pixels. Different from the previous methods, we utilize the statistical correction method in modules. It not only calculates the pixels of the whole image, but also divides the image into four blocks according to the center point for spatial information computation. This method is more reasonable and meticulous and can make full use of the spatial relationship between pixels. The details of the method are as follows.

**Figure 2:**
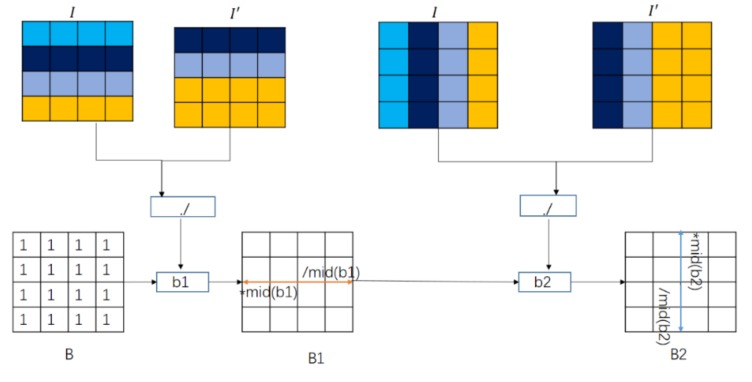
Diagram for calculating the gain coefficients using whole image.

The calculation of the pixels in the whole image is shown in Figure 2. In the vertical direction, the method first translates the original image one line upward, and then copies the original pixel value in the last line to get a new image $I^{\prime }$. It then divides the original image and the new image point by point to get the coefficient $b_{1}$, which represents the similarity ratio between the current pixel and its neighbor pixel. Assuming that the initial value of gain B is an all-one matrix, taking the center point as the reference point, all pixels on its left are multiplied by the median value of $b_{1}$, and all pixels on its right are divided by the median value of $b_{1}$, and thus the gain $B_{1}$ is obtained. The same operation is performed in the horizontal direction to get the similarity ratio $b_{2}$ between adjacent pixels, and then $B_{2}$ is obtained with $B_{1}$ and $b_{2}$. All the pixels above the center point are multiplied by the median of $b_{2}$, and then all pixels below the center point are divided by the median of $b_{2}$.

**Figure 3:**
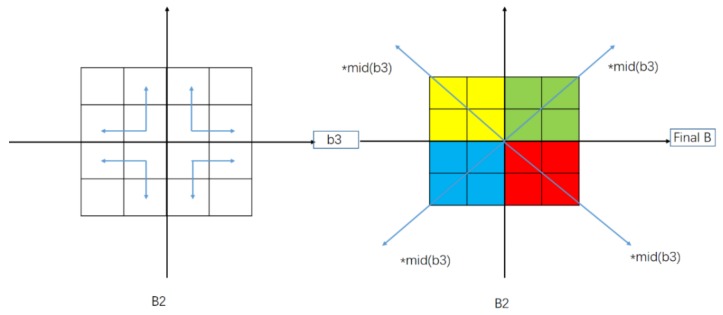
Diagram of modular pixel consistency calculation.

The calculation between adjacent pixels in modules is shown in Figure 3. In this part, the whole image is divided into four blocks based on the center point, and the operations shown in Figure 2 are performed on each block. Then, the similarity ratios of four adjacent pixels are combined to get $b_{3}$. From the center point, the median value of $b_{3}$ is multiplied by the top-left, bottom-left, top-right, and bottom-right, respectively. Finally, the gain correction coefficient B is obtained.

### 3.2. Interframe Registration NUC with Statistical Gain

Apart from considering the spatial information, we also integrate the interframe registration NUC method into our approach. The gain coefficient obtained as shown in Section 3.1 is set as the initial value of the inter-frame registration NUC method, which can increase the speed and accuracy of the convergence.

Since the application of this paper is long range sky background and the optical focal length is relatively long, the images used in the correction method have little high frequency information. Thus, phase correlation [24] is used to detect the relative displacement between two images with the same content. In the registration NUC approach, the phase correlation is utilized for motion estimation, while cross correlation is used for subpixel image registration.

Firstly, the two inter frame images $I_{1}$ and $I_{2}$ need to be converted into the Fourier domain, $I_{1}^{\prime }$ and $I_{2}^{\prime }$, thus the correlation between frames can be calculate as

(15) $$C=\frac{I_{1}^{\prime }*I_{2}^{\prime }{}^{*}}{\left|I_{2}^{\prime }*I_{1}^{\prime }{}^{*}\right|}$$

where the parameter C can be seen as a map representing the correlation information between frames. Using C, we can confirm whether the image pair is valid or not,

(16) $$V\left(I_{1}{,I}_{2}\right)=\left\{\begin{array}{c} 1,\max\left(C\right)>\mathrm{mean}\left(C\right)*20\\ 0,\mathrm{otherwise} \end{array}\right.$$

If the peak of the map C is greater than the mean of C, we consider this pair of frames as a valid pair and a registration is to be done. The location of the peak of C is found as (x,y) for motion estimation, and then the super pixel registration accuracy is refined in a 5 × 5 window,

(17) $$C_{\mathrm{new}}\left(x-d\text{:}x+d,y-d\text{:}y+d\right)=C(x-d\text{:}x+d,y-d\text{:}y+d)$$

which builds a phase correlation mask. The mask is multiplied by the image $I_{2}^{\prime }$ to obtain the registered image $I_{2}^{\prime \prime }$.

(18) $$I_{2}^{\prime \prime }{=I}_{2}^{\prime }*C_{\mathrm{new}}$$

$I_{2}^{\prime \prime }$ is converted from the Fourier domain back to sequential domain as $I_{2}^{\mathrm{reg}}$. Finally, the objective error could be built as

(19) $${e=(I}_{1}{-I}_{2}^{\mathrm{reg}}{)}^{2}$$

Then, Equations (8) and (9) are used to iteratively converge to the optimal gain and offset coefficients to achieve NUC. Unlike the usual iterations, the initial gain is estimated in advance by the pixel consistency strategy, which could effectively improve the NUC performance.

## 4. Experimental Results and Comparative Study of Related Methods

In this section, the proposed SIR method is compared with two representative NUC techniques: two-point NUC method [12] and IRLMS method [20]. The two-point method is simple but effective. It is one of the most widely used correction methods in engineering; it could always generate excellent correction images. In addition, IRLMS is a representative registration-based correction method at present. Our method is proposed on the basis of this method, but achieves better correction performance. The detailed features of the tested IR system are given in Table 1.

### 4.1. Evaluation

**Figure 4:**
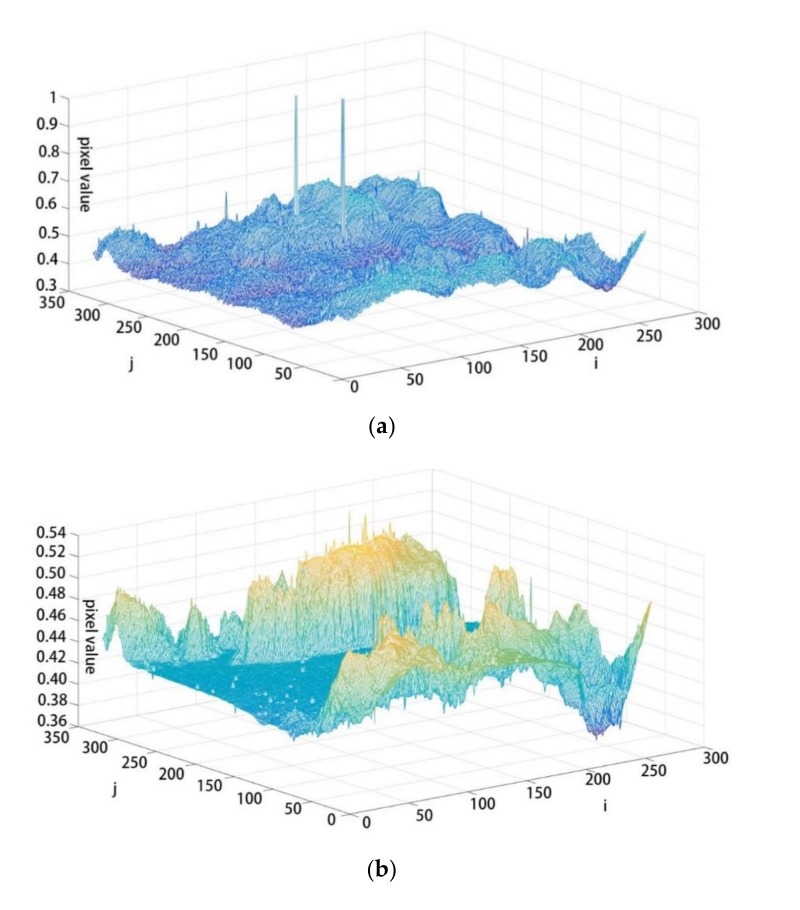

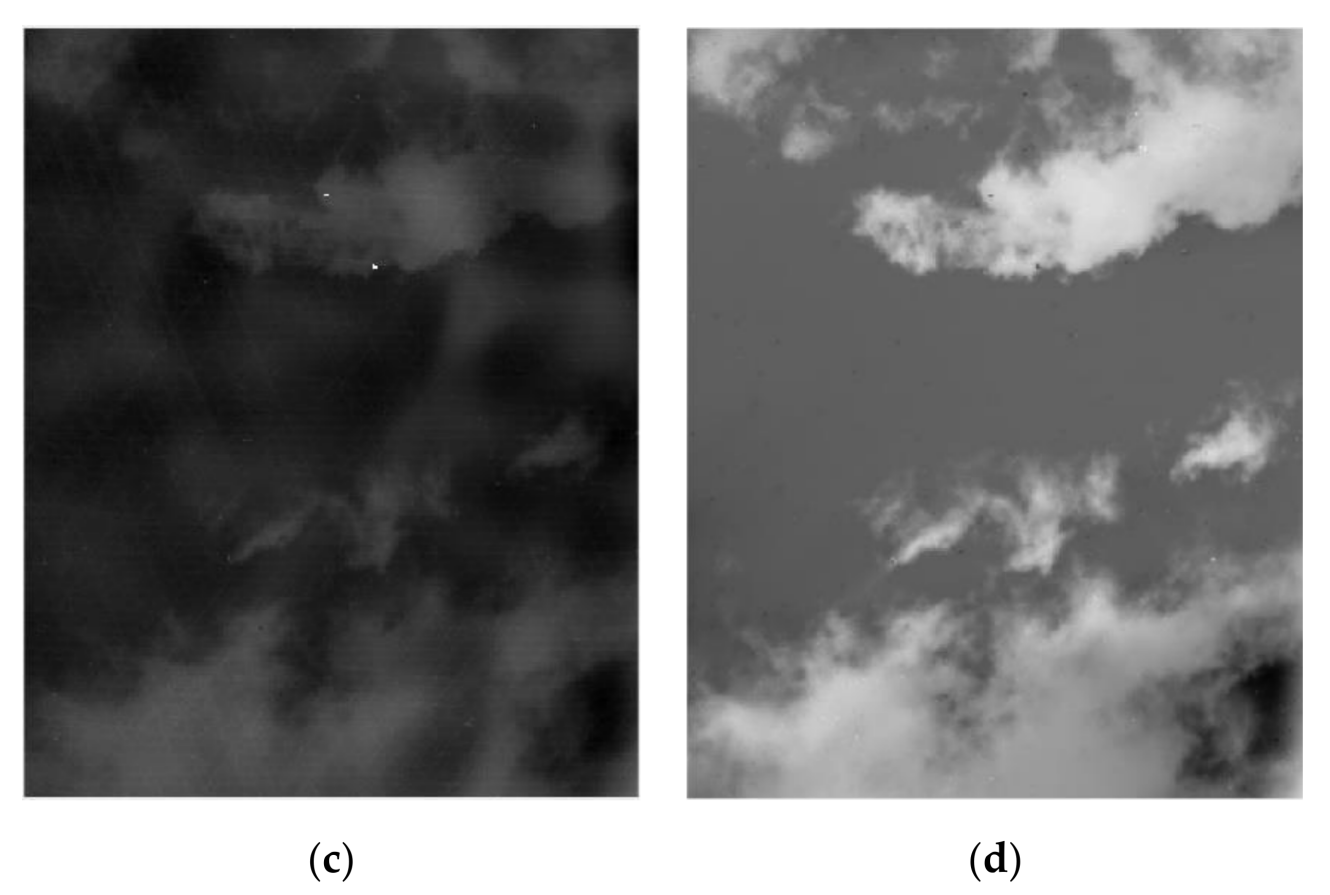
Pixel value distribution contrast map before and after CSIR NUC. (**a**) pixel value distribution contrast map before CSIR; (**b**) pixel value distribution contrast map after CSIR; (**c**) corresponding picture before CSIR; (**d**) corresponding picture after CSIR.

It is known that reducing the standard deviation of the image can improve the signal-to-noise ratio of the target and provide help for detection, by reducing false alarms or detecting longer distances. Thus, the global and local mean standard deviations were used to quantitatively evaluate the NUC performance on real infrared sequences. We tested the represented approach and our approach on three real infrared sequences collected by ourselves. Figure 4 shows the image and its pixel distribution before and after correction.

In Figure 4a,c, it is difficult to distinguish the content of the image due to the non-uniformity of the focal plane before correction, and there are two blind elements. However, by using the proposed method, we can easily determine the position of foreground clouds in the image, and the blind elements disappeared.

The results of the global mean standard deviation on each sequence are shown in Table 2 using Equation (20). As can be seen in Table 1, the average standard deviation of our method is the smallest. The other methods are the two-point method (that uses black body to achieve results), the statistical method, the IRLMS method (that uses consistent w (all-one) and b (all-zero)), the proposed method (CSIR), and the registration method using the two-point result as its initial value. Significantly, we use the average value of IRLMS results, CSIR results, and NSIR results, which were calculated from previous pictures, to correct the latest pictures.

(20) $$\mathrm{GSTD}=\;\sqrt{\frac{1}{N}\sum_{i=1,j=1}{\left(x_{i}-\mu \right)}^{2}+{\left(y_{j}-\mu \right)}^{2}}$$

These results also show that statistical methods have better accuracy in the two-picture sequence because they use the scenario itself to correct the result. However, in Sequence 2, statistical methods ahd poor performance, which was caused by flash pixel, while the CSIR method could improve the accuracy of the statistical methods in these three sequences.

Table 3 lists the frame numbers required by the three methods to achieve the above results. It should be noted that, when frame number grew three-fold or more, IRLMS and NSIR could have better results. However, this paper is mainly focused on low delay time NUC correction method, thus we do not care about situations that need too many frames.

For a more detailed comparison, we also evaluated our method on the local mean standard deviation using Equation (21). By cropping the same position of the corrected image and calculating the average standard deviation of the area, we compared the corrective effect of different methods on the details. The results of the local mean standard deviation over each sequence are presented in Table 4. Our method still performed well. Significantly, the frame numbers of the three methods are the same in Table 4.

(21) $$\mathrm{LSTD}=\;\sqrt{\frac{1}{5*5}\sum_{i=1,j=1}{\left(x_{i}-\mu \right)}^{2}+{\left(y_{j}-\mu \right)}^{2}}$$

### 4.2. Non-Uniformity Correction with Time Variation in Different Scenes

To verify more clearly the advantage of low delay time (corresponding to frame number) for proposed method, we used three different scenario pictures and different frame numbers to test the two-point method, the IRLMS method, and the CSIR method.

**Figure 5:**
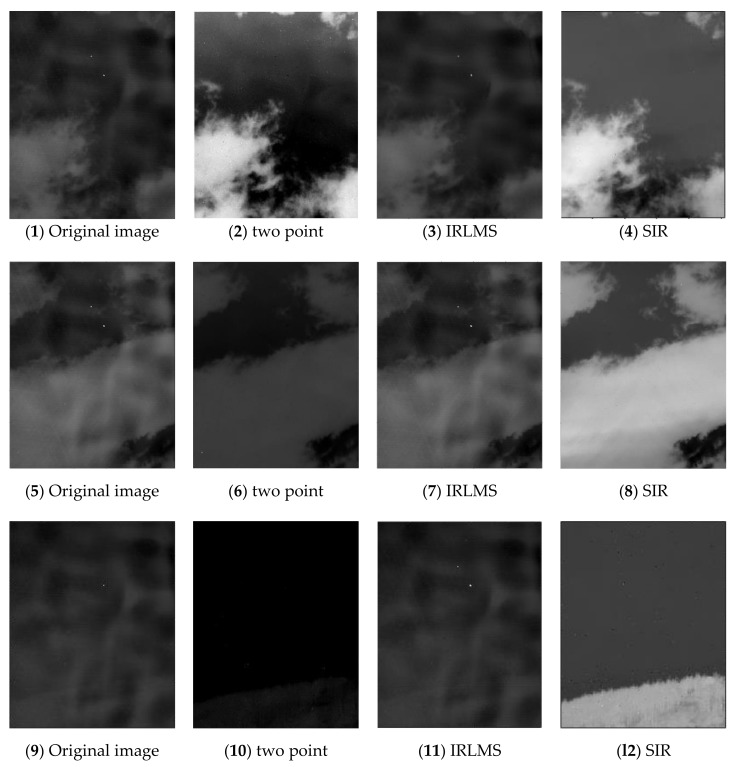
NUC performance comparison of representative frame of the three test sequences: (**1**,**5**,**9**) original images; (**2**,**6**,**10**) two-point method; (**3**,**7**,**11**) IRLMS method; and (**4**,**8**,**12**) proposed SIR method.

Figure 5 shows the original pictures and the results of two-point correction, IRLMS correction using 150 frames and CSIR correction using 150 frames. As shown in the figure, the field of view in the first two sequences is cloud, and the field of view of the third sequence is forest. For the two-point method, although the non-uniform noise and ghost are probably removed from the corrected image, the image noise is serious from the visual effect. The noise mainly comes from the error of the linear model approximated to the nonlinear model. The dynamic temperature range based on the scene is large. For pixels far from the reference temperature point, the correction error increases gradually.

The IRLMS method, in contrast, shows poor performance when using relatively few pictures in the sequence. In this case, the IRLMS method can hardly remove the non-uniform noise. With the continuous iteration of IRLMS, the influence of non-uniformity on the image can be gradually eliminated. However, convergence time is important in many real-time situations. In such cases, this method converges slowly, and cannot be used.

**Figure 6:**
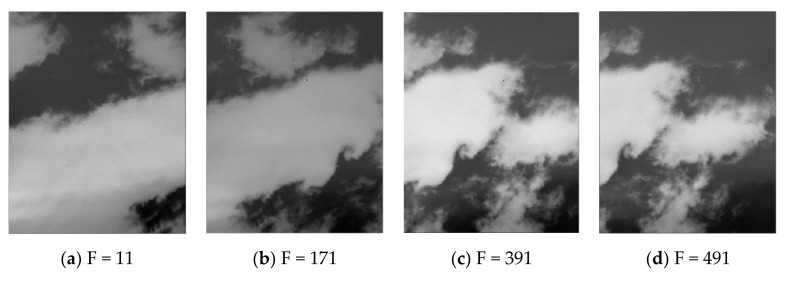
Corrected images corresponding to different F frames.

In contrast, the proposed method has the best visual effect. With this method, the effect of non-uniformity of focal plane can be corrected stably and clearly. At the same time, with the combination of inter-frame registration method, with the iteration of video sequence, there is no ghost or tail phenomenon during the correction process, as shown in Figure 6. These results also prove solidly the necessity of combining the statistical and registration methods.

## 5. Conclusions

In this paper, a novel statistical scene-based non-uniformity correction method with interframe registration is proposed. This method innovatively combines the registration-based method and the statistics-based method to realize NUC. Statistical-based correction methods are mainly used to obtain gain correction coefficients. We make full use of the spatial relationship between the pixels based on modular pixel consistency method. This method provides great help for the final correction by providing appropriate initial values for registration method, thus reducing convergence rate and improving NUC correction accuracy. Detailed experiments demonstrated our great performance. However, there are still some shortcomings to our approach: when flash pixel exists, the registration method is not good enough to have good NUC correction performance. In the future, we will combine more machine learning algorithms to improve the NUC performance and achieve better performance when flash pixel exists.

## Figures and Tables

**Table 1:** Detailed features of the tested IR system.

Parameters	Value
Manufacturer	Sofradir
Spectral band pass (μm)	7.7–11.3
Array size	320 × 256
Pixel size (μm)	30
NETD (mK)	17.32
ADC resolution (bit)	14
Operating temperature (°C)	−196
Full frame rate (f/s)	100
Focal length (mm)	38
F/#	2
Integration time (μs)	300

**Table 2:** Global mean standard deviation (${\times 10}^{-2}$) results for the three test sequences.

	Sequence 1	Sequence 2	Sequence 3
Original image	4.21	6.29	2.85
Two points	4.05	5.01	1.23
Statically method	3.91	6.03	0.79
IRLMS	4.07	6.01	2.63
CSIR	3.88	4.94	0.54
NSIR	4.02	5.34	1.05

**Table 3:** Frame number of the three methods for the three sequences.

	Sequence 1	Sequence 2	Sequence 3
IRLMS	1000	1000	1000
CSIR	200	200	200
NSIR	500	500	500

**Table 4:** Local mean standard deviation (${\times 10}^{-2}$) results for the three test sequences.

	Sequence 1	Sequence 2	Sequence 3
Original image	3.73	6.05	2.63
Two points	3.38	5.00	0.92
Statically method	3.32	5.51	0.98
IRLMS	3.58	5.94	2.60
CSIR	2.83	5.31	0.48
NSIR	3.01	4.98	0.84
